# Increasing socioeconomic disparities in sedentary behaviors in Chinese children

**DOI:** 10.1186/s12889-019-7092-7

**Published:** 2019-06-13

**Authors:** Wei-Jie Gong, Daniel Yee-Tak Fong, Man-Ping Wang, Tai-Hing Lam, Thomas Wai-Hung Chung, Sai-Yin Ho

**Affiliations:** 1 School of Nursing, University of Hong Kong, 4/F William MW Mong Block, 21 Sassoon Road, Pokfulam, Hong Kong, China; 2 School of Public Health, University of Hong Kong, 2/F Patrick Manson Building (North Wing), 7 Sassoon Road, Pokfulam, Hong Kong, China; 3 Student Health Service, Department of Health, 4/F Lam Tin Polyclinic, 99 Kai Tin Road, Kwun Tong, Kowloon, Hong Kong, China

**Keywords:** Sedentary behavior, Health disparity, TV viewing, Video game playing

## Abstract

**Background:**

Sedentary behaviors are prevalent in Chinese children, however, the studies on their trends and socioeconomic disparities are scarce. We examined the time trends of daily television (TV) viewing and video game playing and the associated socioeconomic factors in Chinese children in Hong Kong, the most developed and westernized city in China.

**Methods:**

In a panel data study involving 538,300 primary four and 510,294 primary six students from 1999/2000 to 2008/09, data on socioeconomic status, sedentary behaviors (TV viewing and video game playing) and other lifestyle habits were collected using a self-administered questionnaire. Trends in sedentary behaviors over time were assessed. Their socioeconomic disparities were examined by interactions in generalized estimating equations with the adjustment for weight status and extracurricular physical activities.

**Results:**

The age and sex-standardized prevalence of ≥2 h daily TV viewing decreased from 51.4% (95% confidence interval [CI] 51.1–51.8%) in 1999/2000 to 43.8% (95% CI 43.4–44.2%) in 2008/09 (P for trend < 0.001), whereas that of ≥1 h daily video game playing increased from 8.2% (95% CI 7.9–8.4%) to 22.4% (95% CI 22.0–22.7%). Both sedentary behaviors were more prevalent in boys than girls, but the disparities decreased over time (Ratio of odds ratio [ROR] = 0.996 and 0.924 for TV viewing and video game playing, respectively). In contrast, both sedentary behaviors were increasingly more prevalent in children whose parents had lower education levels or non-managerial/professional occupations (ROR 1.006–1.082).

**Conclusions:**

Children in lower socioeconomic families in Hong Kong were increasingly at risk of having sedentary behaviors over years and thus deserve more attention. Effective strategies targeting children and/or their parents of lower socioeconomic status are needed to reduce sedentary behaviors.

## Background

Sedentary behavior is associated with various unhealthy behaviors, such as irregular sleep [1], disordered eating [2], and adverse health outcomes including obesity, cardiovascular diseases, musculoskeletal disorders, and all-cause mortality [3–5]. Sedentary behavior is defined as any waking behavior characterized by an energy ≤1.5 metabolic equivalents, while in a sitting, reclining or lying posture [6]. School-aged children in the United States (US) and Canada spend over 6 h every day on sedentary behaviors [7, 8]. Recreational screen use, typically TV viewing and computer or video game playing, are regarded as children’s most common sedentary pursuits [8, 9].

TV viewing in Western and Eastern children has shown different trends over time. In 30 Western countries, children’s mean daily TV viewing time decreased slightly from 2.71–3.54 h/day in 2002 to 2.31–3.09 h/day in 2010 [10]. In the US, children’s daily TV viewing time decreased from 2.21 h/day in 2001 to 2.07 h/day in 2011 [11]. In mainland China, the TV viewing time increased from 1.00 h/day in 1997 to 1.43 h/day in 2004 [12], and remained relatively stable during 2004–2011 [13]. Computer and video game playing has increased consistently over the years among both Western and Asian children [10, 13–15].

Children’s sedentariness is associated with their socioeconomic status (SES) [16], and this association differs across countries. In low-income countries, children from a higher SES family have more sedentary time, whereas the pattern is the opposite in high-income countries [17]. With rapid development of economy and community, whether the association between children’s sedentary behaviors and SES changed over time has still not been adequately assessed. Ignoring socioeconomic disparity would lead to erroneous estimation of children’s potential needs for targeted interventions, which might interfere with the benefits of disadvantaged youth who remain at a higher risk of sedentary-related problems, such as unfavorable body composition, cardiometabolic syndrome and problematic behaviors [9].

As one of the most developed and westernized cities in Asia, Hong Kong has exhibited an increasing disparity in income over the past two decades. The Gini coefficient was seen to increase from 0.451 in 1981 to 0.537 in 2011, which is among the highest in the world [18]. This provides an opportunity to examine how SES has affected sedentary behaviors in children. Using a large representative sample, this study was to estimate the time trends in two typical sedentary behaviors, TV viewing and video game playing, and their associations with SES in Hong Kong primary school children.

## Methods

### Study design

This panel data study was population-based. Primary 4 (US equivalent grade 4, P4) or primary 6 (US equivalent grade 6, P6) students during the academic years of 1999/2000 to 2008/09 were included. The study protocol was approved by The Institutional Review Board of The University of Hong Kong/Hospital Authority Hong Kong West Cluster and The Department of Health Ethics Committees.

### Data collection

The administrative data were obtained from the Student Health Service (SHS) of the Department of Health in Hong Kong. A voluntary free territory-wide annual health assessment programme was provided for primary and secondary school children in 12 SHS centres that covered all local regions. Measurements of the children were assessed at the time they attended the service at a designated SHS centre biennially from P4. Each child carried a unique identification number to be tracked and had their identity kept anonymous. The participation rate of SHS was around 83.4% [19]. Details of the survey have been reported elsewhere [20]. We sampled the data of all participated children in Hong Kong from 1999/2000 to 2008/09. Children with incomplete records on the variables utilized in this analysis were excluded.

A self-administered health assessment questionnaire with 20 close-ended lifestyle-related questions was completed by children. TV viewing time was assessed by the question ‘On average, the number of hours I spend on viewing TV each day is’ with four options including ‘less than one hour’, ‘one to two hours’, ‘two to four hours’ and ‘more than four hours’. Video game playing time was assessed by the question ‘My habit of playing video game or computer games is’ with four options including ‘at least one hour every day’, ‘less than one hour every day’, ‘occasionally’ and ‘never’. Previous studies have found that, for children, ≥ 2 h daily TV viewing was associated with reduced physical and psychosocial health [21], while < 1 h daily video game playing was associated with positive psychosocial health [22]. Accordingly, we dichotomized the daily duration of TV viewing into ‘< 2 hours’ and ‘≥ 2 hours’, and video game playing into ‘< 1 hour’ and ‘≥ 1 hour’. In addition, children self-reported their frequency and duration of aerobic exercise after school, which were assessed as < 3 times per week/ ≥ 3 times per week, and < 60 min per week/ ≥ 60 min per week, respectively.

Data on age, sex, study grade, SES, weight, and height were available in the SHS database. Children’s SES was assessed using two separate indicators, the highest education level (primary/below, secondary, and tertiary) and the occupation status (unemployed, manual job, clerical job/service industry, and managerial/professional positions) of parents [20]. Weight was measured by electronic weighing scales to the nearest 0.1 kg, with each child wearing light clothing and no shoes, and height was measured to the nearest 0.1 cm by having the child stand next to a stadiometer without shoes. All measurements were taken by well-trained healthcare workers or nurses according to standard protocols. Body mass index (BMI) was derived by using weight (kg) / height (m)^2^, and weight status was defined using age and sex-specific BMI references according to the International Obesity Task Force (IOTF) Standards [23].

### Statistical analysis

To detect possible bias, the characteristics of the included participants were compared with those excluded with incomplete records. Cohen’s effect size was used to examine the extent of difference between groups, with values < 0.2 as small effect sizes indicating minimal differences [24]. We assessed sedentary behaviors by daily TV viewing and daily video game playing. In order to remove the influences of the possibly changing age and sex distributions over the years, the raw data were standardized by the age and sex distribution of students studying P4 and P6 in 2016/17. Cochran-Armitage trend tests were performed to assess whether the percentage of changes in sedentary behaviors was associated with academic years. Linear models with the regression term of academic years were used to estimate the annual percentage change (APC) of prevalence with 95% confidence intervals (CI).

To account for the extra-covariance of repeated measurements from students in their P4 and P6 as well as those repeating a study year, generalized estimating equations (GEE) were used to investigate how the trends of sedentary behaviors were associated with SES over time. Two groups of models were estimated. Model I examined the effects of academic year, sex, grade, highest parental education and occupation on ≥2 h daily TV viewing or ≥ 1 h daily video game playing after adjusting weight status and the frequency and duration of extracurricular physical activities [25–27]. The adjusted odds ratios (AORs) for academic years were used to determine the overall trends in sedentary behaviors over time. AOR for academic year > 1 indicates that the percentage of sedentary behavior increases in subgroups over the years. Model II included all variables in Models I and also their interactions with academic years for assessing the disparity of academic year trends by different SES subgroups. The ratio of odds ratios (RORs) for the interaction effects were reported [28], ROR > 1 indicates that the difference between groups increases over the years.

We repeated the main analysis by using multiple imputation to handle missing data. Specifically, we imputed missing data by Markov Chain Monte Carlo for 3 times. All analyses were conducted in the Statistical Analysis System (SAS Institute, Cary, NC) version 9.4 using a two-tailed significance level of 0.05.

## Results

Totally 577,260 records from P4 students (9.5 ± 0.59 years) and 536,874 records from P6 students (11.5 ± 0.60 years) were extracted from the original database and, in total, 538,300 (93.3%) P4 students and 510,294 (95.0%) P6 students with complete records remained for the analysis. The characteristics of the included children were compared with those of the excluded children, and Cohen’s effect sizes for the differences in age, BMI, sex, grade, parental education and occupation, TV viewing and video game playing were 0.09, 0.04, 0.05, 0.18, 0.06, 0.08, 0.02 and 0.03, respectively, which were small (< 0.20) [24]. The included participants covered 75.7% P4 students and 66.6% P6 students in the corresponding enrolment in Hong Kong [29].

The weighted demographic characteristics by academic year were summarized in Table 1. There were slightly more boys than girls over the study period. From 1999/2000 to 2008/2009, parents with tertiary educational level increased from 9.7 to 19.7%, whereas those with a manual job decreased from 44.4 to 29.6%.

**Table 1:** Standardized characteristics of primary 4 and primary 6 students by academic year

Characteristics (%)	1999/2000 (*N* = 93,027)	2000/01 (*N* = 98,137)	2001/02 (*N* = 106,228)	2002/03 (*N* = 106,832)	2003/04 (*N* = 115,628)	2004/05 (*N* = 115,498)	2005/06 (*N* = 114,335)	2006/07 (*N* = 111,645)	2007/08 (*N* = 97,955)	2008/09 (*N* = 89,309)
Grade										
P4	50.4	51.3	51.3	50.9	50.7	50.9	50.4	51.1	49.9	49.3
P6	49.6	48.7	48.7	49.1	49.3	49.1	49.6	48.9	50.1	50.7
Sex										
Girls	48.5	48.6	48.5	48.2	48.4	48.3	48.3	48.4	48.6	48.5
Boys	51.5	51.4	51.5	51.8	51.6	51.7	51.7	51.6	51.4	51.5
Highest parental education										
Tertiary	9.7	10.3	10.9	11.8	12.8	14.3	15.5	16.9	18.5	19.7
Secondary	70.7	70.3	70.5	70.1	71.0	71.2	71.6	72.0	71.7	71.6
Primary/below	19.7	19.4	18.6	18.1	16.3	14.6	12.9	11.0	9.9	8.7
Highest parental occupation										
Managerial/professional	21.5	21.3	21.3	21.1	21.8	22.4	23.5	23.5	24.3	24.4
Clerical/service industry	29.8	30.4	31.2	32.0	33.8	35.1	36.8	38.6	39.6	40.9
Manual job	44.4	43.5	42.2	41.2	38.6	36.7	33.9	32.2	30.9	29.6
Unemployed	4.4	4.8	5.3	5.7	5.9	5.8	5.8	5.7	5.3	5.1
Weight status										
Underweight	15.5	15.5	15.7	14.4	14.0	14.1	14.0	13.4	12.3	11.3
Normal	64.6	64.5	64.7	65.1	65.6	65.4	64.6	64.5	64.1	64.1
Overweight	16.2	16.2	16.0	16.5	16.4	16.5	17.1	17.7	18.6	19.2
Obesity	3.7	3.8	3.6	4.0	4.1	4.0	4.3	4.4	5.0	5.3
Age of P4 (Mean ± SD)	9.5 ± 0.49	9.5 ± 0.51	9.5 ± 0.50	9.5 ± 0.49	9.4 ± 0.47	9.4 ± 0.46	9.4 ± 0.46	9.4 ± 0.47	9.4 ± 0.49	9.4 ± 0.50
Age of P6 (Mean ± SD)	11.5 ± 0.56	11.5 ± 0.57	11.5 ± 0.57	11.5 ± 0.57	11.5 ± 0.55	11.5 ± 0.53	11.5 ± 0.50	11.5 ± 0.49	11.5 ± 0.49	11.5 ± 0.49

**Fig. 1:**
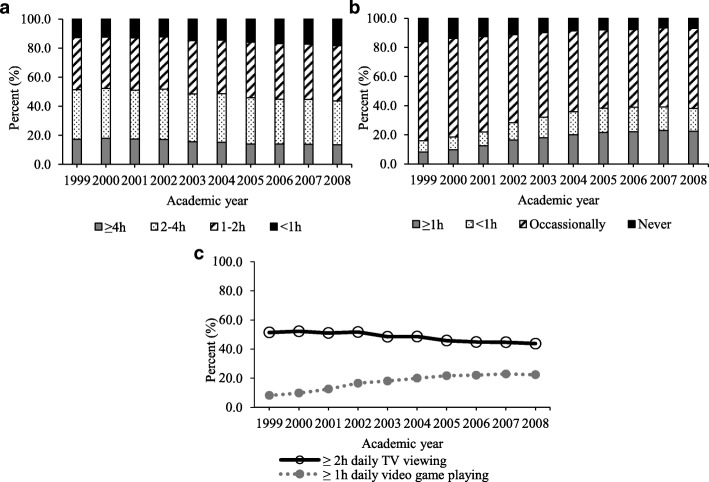
Age and sex-standardized prevalence of sedentary behaviors, including TV viewing by 4 categories (**a**), video game playing by 4 categories (**b**) and ≥ 2 h daily TV viewing and ≥ 1 h daily video game (**c**)

The age and sex-standardized prevalence of the two sedentary behaviors was shown in Fig. 1. From 1999 to 2000 to 2008/09, the prevalence of < 1 h daily TV viewing gradually increased from 12.7% (95% CI 12.5 to 13.0%) to 18.3% (95% CI 18.0 to 18.6%), but that of 1-2 h, 2-4 h and ≥ 4 h decreased from 35.8% (95% CI 35.5 to 36.2%) to 38.0% (95% CI 37.6 to 38.3%), from 34.1% (95% CI 33.7 to 34.5%) to 30.3% (95% CI 29.9 to 30.6%) and from 17.3% (95% CI 17.0 to 17.6%) to 13.5% (95% CI 13.2 to 13.8%), respectively. The prevalence of ≥2 h daily TV viewing decreased from 51.4% (95% CI 51.1 to 51.8%) to 43.8% (95% CI 43.4 to 44.2%), corresponding to APC = − 1.03 (95% CI − 1.29 to − 0.77) (Test for trend: *P* < 0.001). After the adjustment for other factors, the AOR for one later academic year was 0.971 (95% CI 0.969 to 0.972). In contrast, the prevalence of never and occasionally playing video game decreased from 16.0% (95% CI 15.8 to 16.3%) to 6.9% (95% CI 6.7 to 7.1%) and from 68.1% (95% CI 67.8 to 68.5%) to 55.1% (95% CI 54.5 to 55.4%), and that of < 1 h and ≥ 1 h daily video game playing increased steadily from 7.7% (95% CI 7.5 to 7.9%) to 15.7% (95% CI 15.4 to 16.0%) and from 8.2% (95% CI 8.0 to 8.4%) to 22.4% (95% CI 22.0 to 22.7%), respectively. The corresponding APC of ≥1 h daily video game playing was 1.73 (95% CI 1.28 to 2.17) (Test for trend: *P* < 0.001), with an AOR for each later academic year of 1.151 (95% CI 1.148 to 1.157).

### TV viewing

**Fig. 2:**
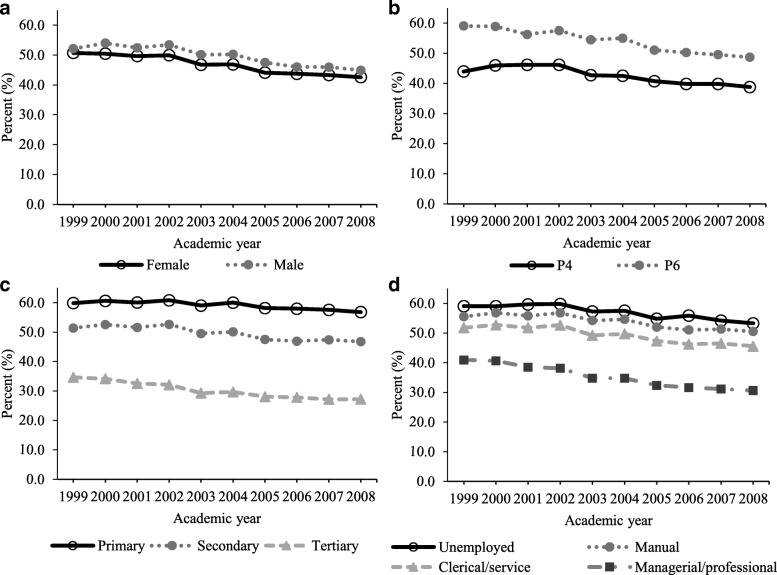
Prevalence of ≥2 h daily TV viewing by socio-demographics. Age and sex-standardized prevalence by sex (**a**), grade (**b**) and highest parental education (**c**) and occupation (**d**)

Figure 2 shows the decreasing prevalence trends of ≥2 h daily TV viewing by sex, grade and SES (Test for trend: all *P* < 0.001). Table 2 shows that throughout the study period, those with higher percentage of ≥2 h daily TV viewing were boys (AOR: 1.133), were studying P6 (AOR: 1.559), had lower parental educational levels (AOR: 1.726 to 2.251), or had parents working at non-managerial/professional levels (AOR: 1.393 to 1.707). Although children in all socio-demographic subgroups showed a decreasing trend of TV viewing, the significant interaction effects with academic year showed the decreasing rates in subgroups were significantly different (Fig. 2). Specifically, the annual risk reduction was slightly greater in boys (AOR: 0.968) than in girls (AOR: 0.971). A larger significant trend difference was found between P6 (AOR: 0.962) and P4 (AOR: 0.978) students. For both, the disparity was narrowing in later academic years. On the other hand, the annual risk reduction was significantly larger in children whose parents with higher education levels or had managerial/professional occupations (Table 2). The disparity among children having different parental education and occupation levels was widening in later academic years.

**Table 2:** Associations of children’s characteristics with ≥2 h daily TV viewing (95% CI)

Characteristics	Annual percentage change^a^	Crude OR	Model I^b^ AOR	Model II^c^	
				AOR for academic year	ROR for the interaction with academic years
Academic year	–	0.976 (0.974,0.977)**	0.971 (0.969,0.972)**	–	–
Sex					
Girls	−1.03(−1.24,-0.82)**	1	1	0.971 (0.969,0.974)**	1
Boys	−1.04(−1.36,-0.71)**	1.116 (1.106,1.125)**	1.133 (1.123,1.143)**	0.968 (0.965,0.971)**	0.996 (0.993,0.999)*
Grade					
Primary 4	−0.84(− 1.18,-0.49)**	1	1	0.978 (0.975,0.980)**	1
Primary 6	−1.26(− 1.52,-1.00)**	1.507 (1.497,1.517)**	1.559 (1.548,1.570)**	0.962 (0.959,0.964)**	0.984 (0.981,0.986)**
Highest parental education					
Tertiary	−0.92(−1.11,-0.72)**	1	1	0.959 (0.954,0.963)**	1
Secondary	−0.70(− 0.98,-0.43)**	2.336 (2.305,2.367)**	1.726 (1.699,1.753)**	0.970 (0.968,0.973)**	1.012 (1.007,1.018)**
Primary/below	−0.40(− 0.58,-0.22)**	3.450 (3.394,3.508)**	2.251 (2.207,2.295)**	0.980 (0.976,0.984)**	1.022 (1.015,1.029)**
Highest parental occupation					
Managerial/professional	−1.28(−1.49,-1.07)**	1	1	0.964 (0.961,0.968)**	1
Clerical/service	−0.87(−1.13,-0.60)**	1.776 (1.756,1.797)**	1.393 (1.375,1.412)**	0.970 (0.967,0.973)**	1.006 (1.002,1.011)*
Manual job	−0.74(−1.01,-0.47)**	2.173 (2.148,2.199)**	1.534 (1.513,1.556)**	0.974 (0.971,0.977)**	1.010 (1.006,1.015)**
Unemployed	−0.72(− 0.97,-0.47)**	2.427 (2.378,2.477)**	1.707 (1.670,1.746)**	0.971 (0.964,0.977)**	1.006 (0.999,1.014)

^a^Tests for trend by Cochran-Armitage trend tests. ^b^Model I included the linear term of academic years and the main effects of sex, grade, highest parental education and occupation, adjusting for weight status and frequency and duration of extracurricular physical activities. ^c^Model II included the variables in Model I and their interactions with academic years. *CI* Confidence interval, *AOR* Adjusted odds ratio, *ROR* Ratios of odds ratios, *ROR* > 1 indicates that the difference between groups increases over the years. *P-value < 0.01, **P-value < 0.001

### Video game playing

**Fig. 3:**
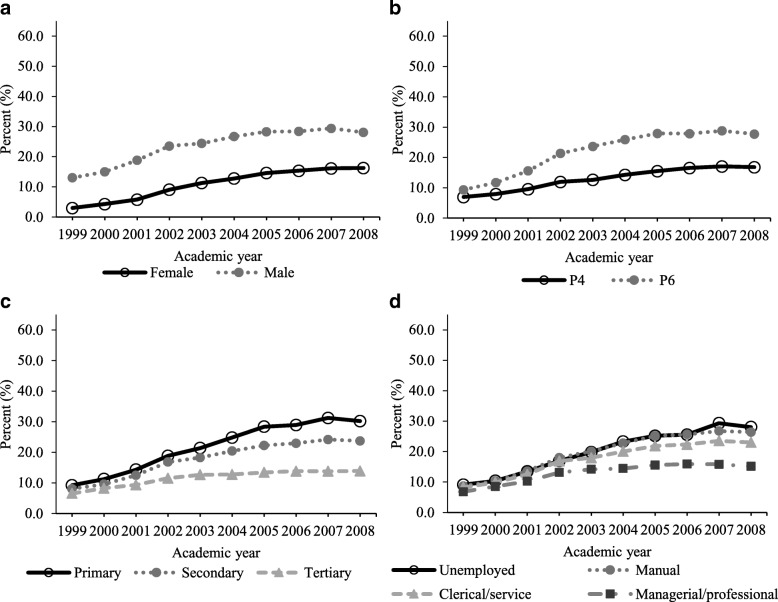
Prevalence of ≥1 h daily video game playing by socio-demographics. Age and sex-standardized prevalence by sex (**a**), grade (**b**) and highest parental education (**c**) and occupation (**d**)

Figure 3 shows the increasing prevalence trends of ≥1 h daily video game playing by all subgroups (Test for trend: all *P* < 0.001). Table 3 shows that throughout the study period, having ≥1 h daily video game playing was associated with being boys (AOR 2.600), studying P6 (AOR 1.918), having lower parental educational levels (AOR 1.432 to 1.695), or parents working at non-managerial/professional levels (AOR 1.251 to 1.411). Although children in all socioeconomic subgroups showed an increasing trend (Fig. 3), there were again significant interactions with academic year (Table 3). Specifically, although the annual percentage change was slightly greater in boys (1.82) than in girls (1.63), the disparity was narrowing in later academic years (ROR 0.924). On the other hand, there was a significantly steeper annual risk increase in children studying P6 and those whose parents had lower parental education levels or non-managerial/professional occupations. The disparity among children in different grades and those having different parental education and occupation levels was widening in later academic years.

**Table 3:** Associations of children’s characteristics with ≥1 h daily video game playing (95% CI)

Characteristics	Annual percentage change^a^	Crude OR	Model I^b^ AOR	Model II^c^	
				AOR for academic year	ROR for the interaction with academic years
Academic year	–	1.155 (1.152,1.157)**	1.151 (1.148,1.153)**	–	–
Sex					
Girls	1.63 (1.33,1.92)**	1	1	1.201 (1.196,1.206)**	1
Boys	1.82 (1.23,2.42)**	2.550 (2.521,2.579)**	2.600 (2.569,2.632)**	1.110 (1.106,1.113)**	0.924 (0.920,0.928)**
Grade					
Primary 4	1.21 (1.00,1.43)**	1	1	1.148 (1.144,1.153)**	1
Primary 6	2.23 (1.52,2.94)**	2.029 (2.009,2.048)**	1.918 (1.899,1.937)**	1.160 (1.156,1.164)**	1.010 (1.006,1.014)**
Highest parental education					
Tertiary	0.81 (0.54,1.08)**	1	1	1.105 (1.098,1.113)**	1
Secondary	1.90 (1.48,2.32)**	1.564 (1.536,1.592)**	1.432 (1.402,1.464)**	1.164 (1.160,1.167)**	1.053 (1.045,1.060)**
Primary/below	2.63 (2.16,3.09)**	1.780 (1.743,1.819)**	1.695 (1.650,1.740)**	1.196 (1.190,1.202)**	1.082 (1.072,1.092)**
Highest parental occupation					
Managerial/professional	0.98 (0.30,1.36)**	1	1	1.138 (1.133,1.144)**	1
Clerical/service	1.79 (1.37,2.21)**	1.465 (1.442,1.488)**	1.251 (1.228,1.274)**	1.149 (1.144,1.153)**	1.010 (1.003,1.015)*
Manual job	2.18 (1.70,2.66)**	1.530 (1.506,1.554)**	1.353 (1.327,1.378)**	1.170 (1.165,1.175)**	1.028 (1.022,1.034)**
Unemployed	2.38 (1.99,2.76)**	1.686 (1.644,1.729)**	1.411 (1.372,1.452)**	1.161 (1.151,1.171)**	1.020 (1.010,1.030)**

^a^Tests for trend by Cochran-Armitage trend tests. ^b^Model I included the linear term of academic years and the main effects of sex, grade, highest parental education and occupation, adjusting for weight status and frequency and duration of extracurricular physical activities. ^c^Model II included the variables in Model I and their interactions with academic years. *CI* Confidence interval, *AOR* Adjusted odds ratio, *ROR* Ratios of odds ratios, ROR > 1 indicates that the difference between groups increases over the years. *P-value < 0.01, **P-value < 0.001

The use of multiple imputation did not materially change the estimated APC of both behaviors and the associations (data not shown).

## Discussion

To our knowledge, this is the first study that has assessed the change of socioeconomic disparities in the trends of sedentary behaviors in children over time. A decreasing annual prevalence of ≥2 h daily TV viewing was accompanied by an increasing annual prevalence of ≥1 h daily video game playing. The disparities in the annual prevalence of both sedentary behaviors by sex and those of ≥2 h daily TV viewing by study grade were found to be diminishing. In contrast, disparities in the annual prevalence of both sedentary behaviors by parental education and occupation were found to be increasing.

The decreasing TV viewing among children appears to be a global trend [30, 31]. According to a representative survey from US children performed in 2015, using TV to view TV programs (71%) was still more than using other devices (15%), such as smartphones and tablets [32]. Hence, whether this decrease comes from lesser use of TV sets or a greater engagement in using other screens is still unknown. Meanwhile, the increase of video game playing was inevitable with its rapidly growing popularity and accessibility. Moreover, our results identified those who were at higher risk of screen time. Boys or children studying at a higher grade tended to spend more time watching TV and playing video games. However, the TV viewing and video gaming differences are narrowing between boys and girls as they progress across the academic years. Nevertheless, the prevalence of ≥1 h daily video game increases in both boys and girls. On the other hand, students in a higher grade would have more access and allowance to own video games they desire, and more cognitive capacity to play and enjoy games as well, which would increase the grade disparity in video game playing.

Our findings are consistent with previous studies reporting that lower SES is associated with unhealthy behaviors in developed societies [33–35]. We further showed increasing disparities in the prevalence of both sedentary behaviors among children by parental education and occupational level during the study period, may have resulted from their highly dependence on their parents for living. Children’s exposures, attitudes and experiences toward accessing media could be influenced by their family environment [35]. As role models, sedentary parents usually result in similarly sedentary children. The disparities could be partially explained by the communication inequality theory, which describes the disadvantages of the lower social classes in how they access, seek, process and act on health information [36, 37]. In developed areas, parents of lower SES may tend to neglect health information from television, radio, newspapers, magazines and the Internet. Thus, they are often insensitive to the adverse impacts of prolonged screen time and less likely to restrict their children from sedentary behaviors [38]. Further studies on the underlying reasons are in need.

Children of lower SES in Hong Kong was associated with higher risk of sedentary behaviors, whereas those in mainland China have been shown to have lower risk or no association with sedentary behaviors [39–42]. The difference of association between the two regions is consistent to the observed difference of that between high-income places including Hong Kong and low-middle-income places including mainland China [17]. Screen devices such as TV and computers are generally more affordable in high-income places than in low-income places. Hence, with lower awareness of the potential hazards of sedentary behaviors in lower SES families, relatively higher accessibility of screen devices in high-income places would result in a positive association between low SES and sedentary behaviors.

Being sedentary in childhood has immediate adverse effects on children’s physical and psychological well-being, which may track into adulthood [43]. It may be difficult to change sedentary behaviors because of their strong habitual component [44]. Our study has addressed the need to prioritize interventions for families of lower SES. However, according to the inverse care law, children’s need is badly matched with their access to preventive interventions [45]. For example, although using electronic screen monitors could effectively reduce children’s TV watching time [46], families of lower SES may be less likely to use them due to financial limitations or lack of knowledge. To narrow SES disparities in children’s sedentary behaviors, more accessible preventions targeting families of lower SES are desirable. For instance, positive reinforcement methods such as goal setting with rewards have been shown to be effective in motivating behavioral changes in children [47, 48]. Moreover, stimulus control methods such as restructuring the environment for screen use have also been proved to be effective in preventing children from engaging sedentary behaviors [47]. Generally, greater family involvement is essential to put these methods into practice [49]. Therefore, enhancing parents’ awareness should be a priority to reduce sedentary behaviors in children from lower SES.

With the rising popularity of portable media devices, more children may shift from viewing TV to viewing the smaller portable media devices. Thus this decreasing trend in TV viewing would continue, and so would the increasing trend in video game playing. Moreover, since the socioeconomic disparities have been attributed to lower health consciousness among the lower social classes, increased affordability of portable media devices will enhance these disparities.

Our study had some limitations worth noting. First, 2.5–4.2% of the P4 and P6 students were international school students, who may not be Chinese although ethnicity was not recorded [29]. However, any effect on the results shall be small as the prevalence was low. Second, we have only considered parental education and occupation as indicators of SES due to the lack of information on household income in the SHS database, however, our results are similar to those using income and composite SES scores in developed societies, depicting the inverse relationship between higher levels of sedentary behaviors and lower SES [50–52]. Third, sedentary behaviors was self-reported by the participants, which may jeopardize the responses due to social desirability and recall bias. However, if the influence of social desirability is similar over the years, then the impact on the time trends would be minimal and our results on social disparities should be intact. Moreover, it would be best to measure the screen time by prospective close observation, but this may unfortunately be infeasible in a large-scale study with follow-up [53]. Last, screen use is only one type of sedentary behaviors. Further studies on other sedentary behaviors, such as doing homework or studying, will be desirable. Due to the setting of the questionnaire, the trend of a total screen time for Hong Kong children could not be given in this study. Instead, we assessed individual types of screen time as they may carry different effects on children’s health [54], and their possible changes could be identified separately.

## Conclusions

Children in lower socioeconomic families in Hong Kong were increasingly at risk of having sedentary behaviors over years and thus deserve more attention. Effective strategies targeting children and/or their parents of lower socioeconomic status are needed to reduce sedentary behaviors.

## Data Availability

The data that support the findings of this study are available from the Student Health Services of the Department of Health in Hong Kong but restrictions apply to the availability of these data, which were used under agreement for the current study, and so are not publicly available. Data are however available from the authors upon reasonable request and with permission of the Student Health Services of the Department of Health in Hong Kong.

## Abbreviations

AOR: Adjusted Odds Ratio
APC: Annual Percentage Change
BMI: Body Mass Index
CI: Confidence Interval
IOTF: International Obesity Task Force
P4: Primary 4
P6: Primary 6
ROR: Ratio of Odds Ratio
SAR: Special Administrative Region
SAS: Statistical Analysis System
SES: Socioeconomic Status
SHS: Student Health Service
TV: Television
US: The United States
